# Protein-Pacing from Food or Supplementation Improves Physical Performance in Overweight Men and Women: The PRISE 2 Study

**DOI:** 10.3390/nu8050288

**Published:** 2016-05-11

**Authors:** Paul J. Arciero, Rohan C. Edmonds, Kanokwan Bunsawat, Christopher L. Gentile, Caitlin Ketcham, Christopher Darin, Mariale Renna, Qian Zheng, Jun Zhu Zhang, Michael J. Ormsbee

**Affiliations:** 1 Human Nutrition and Metabolism Laboratory, Department of Health and Exercise Sciences, Skidmore College, Saratoga Springs, NY 12866, USA; redmonds@skidmore.edu (R.C.E.); cketcham@skidmore.edu (C.K.); cdarin@skidmore.edu (C.D.); mariale.p.renna@gmail.com (M.R.); qzheng1@skidmore.edu (Q.Z.); junzhu.zhang@gmail.com (J.Z.Z.); 2 Integrative Physiology Laboratory, Department of Kinesiology and Nutrition, University of Illinois at Chicago, Chicago, IL 60612, USA; bunsawa2@uic.edu; 3 Department of Food Science and Human Nutrition, Colorado State University, Fort Collins, CO 80523, USA; christoper.gentile@colostate.edu; 4 Florida State University, Institute of Sports Sciences & Medicine, Department of Nutrition, Food and Exercise Sciences, Tallahassee, FL 32304, USA; mormsbee@fsu.edu; 5 Discipline of Biokinetics, Exercise, and Leisure Studies, University of KwaZulu-Natal, Durban 4041, South Africa

**Keywords:** protein-pacing, physical performance, cardiometabolic-risk, PRISE exercise training

## Abstract

We recently reported that protein-pacing (P; six meals/day @ 1.4 g/kg body weight (BW), three of which included whey protein (WP) supplementation) combined with a multi-mode fitness program consisting of resistance, interval sprint, stretching, and endurance exercise training (RISE) improves body composition in overweight individuals. The purpose of this study was to extend these findings and determine whether protein-pacing with only food protein (FP) is comparable to WP supplementation during RISE training on physical performance outcomes in overweight/obese individuals. Thirty weight-matched volunteers were prescribed RISE training and a P diet derived from either whey protein supplementation (WP, *n* = 15) or food protein sources (FP, *n* = 15) for 16 weeks. Twenty-one participants completed the intervention (WP, *n* = 9; FP, *n* = 12). Measures of body composition and physical performance were significantly improved in both groups (*p* < 0.05), with no effect of protein source. Likewise, markers of cardiometabolic disease risk (e.g., LDL (low-density lipoprotein) cholesterol, glucose, insulin, adiponectin, systolic blood pressure) were significantly improved (*p* < 0.05) to a similar extent in both groups. These results demonstrate that both whey protein and food protein sources combined with multimodal RISE training are equally effective at improving physical performance and cardiometabolic health in obese individuals.

## 1. Introduction

Although it is well-accepted that increased protein intake and physical activity are likely effective strategies to combat the rise in obesity [1], there is a paucity of well-controlled lifestyle interventions. Indeed, there is less data available on lifestyle interventions combining increased protein intake above the recommended dietary allowance (RDA) and exercise training in overweight populations that quantify changes in fitness-related performance outcomes such as muscular strength and endurance, flexibility, and balance.

Increasing protein intake above recommended levels has been shown to enhance protein synthesis, postprandial thermogenesis, lean body mass, satiety, and cardiometabolic health [2,3]. We recently demonstrated that, compared to current recommendations (three meals/day and 0.8 g/kg BW/day), a protein-pacing diet (five to six meals/day; three of which were whey protein supplemented and >0.3 g/kg BW/meal; >1.4 g/kg BW/day) elicited greater improvements in body composition and thermic effect of feeding, during both energy balance and deficit in overweight adults [4].

In recent years, increasing attention has been given to healthy lifestyle routines that combine multiple fitness components into one training program or can be delivered with the support of computer-based technologies [5,6]. Indeed, several studies have reported training programs that combine resistance and endurance exercises are more effective at improving body composition and reducing metabolic disease risk than either training modality alone [7,8,9]. In addition to traditional resistance and endurance exercise, nonconventional modalities such as yoga, tai chi, pilates, and interval sprint training have become increasingly popular among the general public [10,11,12].

In light of this popularity of both protein-pacing (P) and combined exercise training, we recently compared the effectiveness of P (six meals/day, three meals/day of which were whey protein) combined with either traditional resistance training or a regimen that included resistance exercise, interval sprint exercise, stretching (yoga, pilates), and endurance exercise (RISE training), and found that PRISE (protein-pacing, resistance, interval, stretching, endurance training) resulted in greater reductions in body weight, total and abdominal (including visceral) fat mass, as well as greater gains in percent lean mass [13]. Collectively, these data provide experimental evidence that a whey supplemented protein-pacing diet (WP) combined with a multi-mode fitness program (RISE) results in greater cardio-metabolic health benefits than other training and dietary regimens, a finding supported by others [14]. Whether this diet-exercise (PRISE) combination also favorably improves indices of fitness-related performance outcomes in this obese/overweight population was a major focus of the current study.

Of practical relevance is whether the source of protein supplementation (whey protein supplemented *vs.* food protein sources only) influences metabolic and physical performance outcomes. Some [15,16], but not all [17], studies have reported that whey protein is more effective at improving body composition and disease risk than other protein sources (e.g., soy, pea, casein). Most intervention studies [18], including those from our own laboratory [4,13,19,20], have primarily used powdered or ready-to-drink whey protein supplements, and few data exist comparing the effects of whey protein supplementation to protein derived from a variety of whole food sources (animal and plant). This gap in the literature limits the general application of existing data as well as a preference among some individuals to consume whole foods rather than protein supplements [21].

With this background, the primary purpose of the present study was to compare the effects of a protein-pacing diet consisting of either whey protein supplementation (WP; consumed as three of the six daily meals) or protein-rich food sources (FP; consumed for all six daily meals) combined with RISE training on fitness-related performance outcomes, as well as cardiometabolic and body composition measures. Given the previously documented benefits of whey protein compared to other protein sources, it is hypothesized that the WP will elicit more favorable changes in performance, cardiometabolic, and body composition outcomes than FP. It is important to note that only protein was adjusted whereas fat and carbohydrate intake were not intentionally modified.

## 2. Materials and Methods

### 2.1. Participants

**Figure 1:**
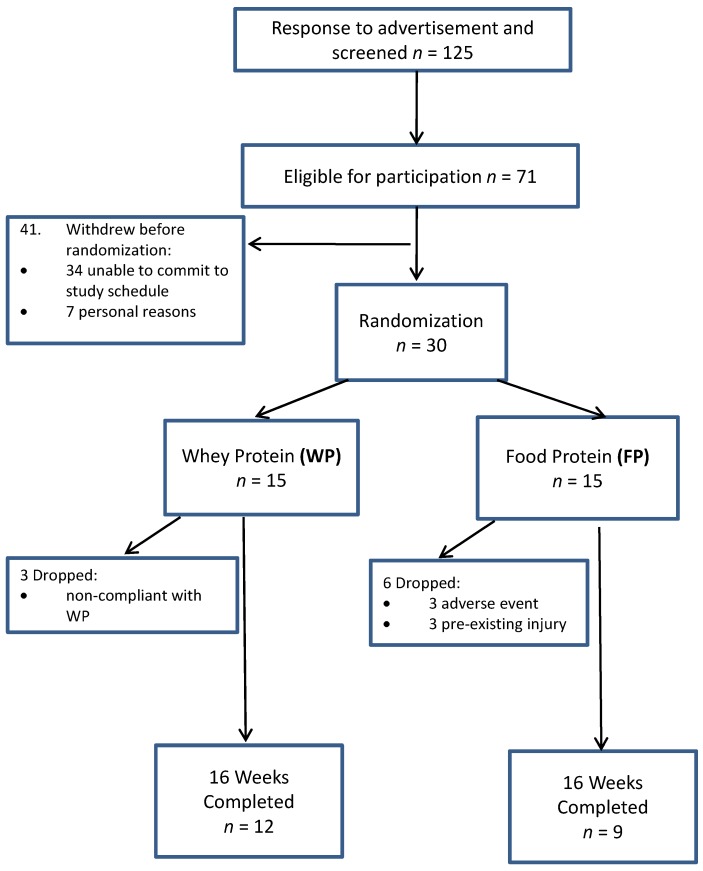
CONSORT (Consolidated Standards of Reporting Trials) flow chart of participants during the study intervention.

A total of 125 individuals from the surrounding Saratoga Springs, NY, USA community responded to flyers and newspaper advertisements and were screened for participation. Of the 71 volunteers who met eligibility criteria, 30 middle aged (50 ± 8 years) men and women started the study (Figure 1).

All participants were nonsmokers; overweight (body mass index, BMI; 25–29.9 kg/m^2^) or obese (BMI > 30.0 kg/m^2^); sedentary (<30 min, two days/week of structured physical activity); weight stable ±2 kg for at least six months prior to beginning the study); and absent of overt cardiovascular or metabolic diseases (e.g., type 2 diabetes, thyroid disease) as assessed by a medical history and physical examination. All procedures were approved by the Skidmore College Institutional Review Board Committee. The nature, benefits, and risks were explained to the volunteers and their written informed consent was obtained prior to participation. This trial was registered at clinicaltrials.gov as NCT02594228.

### 2.2. Experimental Design

Participants were randomized to one of two nutritional intervention groups for the 16-week study (Figure 1): (1) combined exercise training (RISE) and food protein (FP, *n* = 15) or (2) RISE and whey protein (WP, *n* = 15). Both groups were asked to consume five to six small meals each day containing ~20–25 g of a high quality protein source (Supplementary Materials Table S1). Of note, we purposefully did not include a RISE only control group because we were interested in specifically comparing the source of higher protein consumption given the well-established increased dietary protein needs during exercise training [22]. All testing procedures were measured at baseline and following the 16 week intervention period. Participants in the FP group consumed protein only from high quality food sources for each of the meals. The WP group replaced the food protein of three meals on exercise days and two meals on non-exercise days with 20–25 g of whey protein supplement (Classic Whey; Optimum Nutrition). For all meals, participants were provided with a menu of foods from which to choose. Examples included milk, Greek yogurt, eggs, lean meats, fish, poultry, and specific plant sources, including legumes, nuts, and seeds. The number of recommended daily calories to consume was estimated to match the caloric requirements of each individual as measured by resting metabolic rate and measured/estimated physical activity level but was *ad libitum*, and not energy-restricted. Both groups followed the same protocol in terms of the timing of meals: all meals were evenly spaced throughout the day and one meal was consumed within one hour of waking in the morning and another two hours prior to bed. On exercise days, both groups consumed a protein meal (20–25 g) within 60 min after completion of exercise. For WP, they were required to consume this meal as 20–25 g of whey protein giving them a total of three servings of whey on exercise days. For FP, this required a protein-rich food meal of 20–25 g. On non-exercise days, both groups consumed similar amounts of total protein at each of their six meals per day.

### 2.3. Exercise Training

All subjects in both groups participated in the same multiple exercise training regimen as described previously [13]. Briefly, the training program consisted of four specific types of exercise: (1) resistance training; (2) interval sprints; (3) stretching/yoga/pilates; and (4) endurance exercise (RISE training; Supplementary Materials Table S2). Subjects underwent four exercise sessions/week, and the sessions rotated through the four types of exercise, such that each of the four exercises was performed one day/week. To familiarize participants with the individual exercises and to ensure compliance, all training sessions were performed in the Skidmore College Sports Center under the supervision of at least two members of the research team.

Specific details of the four types of exercises that comprise the RISE training have been previously published [10]. Briefly, the resistance (R) training sessions were completed within 60 min and consisted of a dynamic warm-up, footwork and agility, lower and upper body resistance, and core exercises performed at a resistance to induce muscular fatigue in 10–15 repetitions and for two to three sets. A 30 s recovery was provided between sets and a 60 s recovery was allowed between different exercises. The sprint interval (I) training sessions were completed within 40 min and consisted of 5–10 sets of 30–60 s of all-out exercise interspersed with 2–4 min of rest after each exercise. Participants were allowed to perform the sprints using any mode of exercise (treadmill, elliptical machine, stationary bikes, swimming, snowshoeing, cycling, rollerblading, *etc.*). The stretching/yoga/pilates regimen was based on traditional yoga poses with modern elements of pilates training for a total body stretching, flexibility, and strengthening workout. All sessions were completed within 60 min and were led by a certified yoga instructor (PJA). Finally, endurance exercise training was performed for 60 min or longer at a moderate pace (60% of maximal effort). Participants were allowed to choose from a variety of aerobic activities, including walking, jogging, cycling, rowing, swimming, *etc*.

### 2.4. Laboratory Testing Procedures

All testing (see below) was administered pre-intervention (week 0) and post intervention (week 17) between 0600 and 0900, following a 12-h fast and 48-h abstinence from caffeine and alcohol intake, and 48–72 h after the last exercise session to eliminate the acute effects of the last bout of exercise.

### 2.5. Body Weight and Composition

Body weight was obtained during each visit with a standard digital scale (Befour Inc., Cedarburg, WI, USA). Height was measured without shoes using a stadiometer. Waist circumferences were obtained in centimeters with a standard tape measure. Waist measurement was taken at the area with the smallest circumference between the rib cage and the iliac crest and obtained by the same investigator (PJA). Body Composition was assessed by Dual Energy X-ray Absorptiometry (iDXA; Lunar iDXA; GE Healthcare, Madison, WI, USA; analyzed using Encore software version 13.6; GE Healthcare). Total body adiposity, % body fat, lean body mass, appendicular composition, visceral adipose tissue (VAT), and regional abdominal adiposity were all analyzed from DXA scans as previously described [13].

### 2.6. Dietary Intake and Feelings of Hunger and Satiety

Throughout the intervention, subjects maintained a daily food log that included all food and beverages consumed each day, including meal timing. To further verify compliance, food intake was analyzed from a representative three-day period of two week days and one weekend day at weeks 0 and 16 using Food Processor SQL Edition (version 10.12.0, 2012; ESHA Research, Salem, OR, USA) as previously described [13,23]. All dietary analyses were performed by the same technician. Visual analog scales (VAS) were administered at baseline and week 16 to evaluate the effects of the lifestyle interventions on hunger, satiation, and desire-to-eat [13].

### 2.7. Physical Performance Assessments

Measures of one repetition strength of the upper and lower body were assessed via the bench and leg press, respectively, following National Strength and Conditioning Association (NSCA) guidelines, as previously described [20]. Upper body muscular endurance of the chest and abdominals were assessed with push-ups and sit ups completed in one minute, respectively.

For the push-ups, men started in a plank position with arms extended and hands placed under the shoulders and balancing on the toes. A successful push-up was defined as lowering the body so elbows reached 90° followed by a return to the starting plank position. Women started in the plank position balancing on the hands and knees and followed identical procedures as the men. Participants were asked to perform as many push-ups as possible within 60 s in a continuous pattern with no more than two seconds between repetitions.

Sit-ups required participants to start in the supine position with knees bent to 90° and feet flat on the ground and supported by a research team member and arms folded across the chest. A successful sit-up required participants to curl up to a 90° position (vertical) to the floor and then return to the starting position. The sit-up action was continuous, with a single rest of no more than 2 s allowed between repetitions and performed to achieve the maximum number of sit-ups in 60 s.

Postural static standing balance was assessed with the stork balance test. Participants were instructed to balance on the dominant leg with the heel lifted off the ground while the non-dominant leg was bent at the knee to allow the foot to be placed gently against the inside of the dominant knee. Hands were placed on top of the iliac crests. The trial ended when the heel of the dominant leg touched the floor, the hands came off of the hips, or the non-dominant foot was removed from the dominant standing leg. Participants were provided three attempts and the best time was recorded for analysis.

Lower back and hamstring flexibility was assessed with the sit and reach test. This was administered using a standard preassembled metal 30.48 cm by 53.34 cm box (Lafayette Instrument Company, Lafayette, IN, USA). Participants sat on the floor with legs extended and shoulder width apart with feet flat against the front side of the box and arms fully extended with one hand over the over on the top edge of the box beginning at the zero point. When prompted, they flexed forward from their waist with legs straight and slowly slid their hands along a ruler positioned on top of the box. Following three attempts, the maximal distance traveled with their hands was recorded.

Hand grip strength using a dynamometer (Lafayette Instruments, Model 78011) was performed in the standing position with 90 degrees of elbow flexion. Participants were allowed three trials using the dominant arm only and the highest value was recorded.

### 2.8. Cardiometabolic Biomarkers

A 12-h fasted venous blood sample was obtained in an ethylenediamine tetraacetic acid (EDTA)-coated vacutainer tube and centrifuged for 15 min at 4 °C. Plasma leptin, adiponectin, and insulin concentrations were determined using commercially available ELISA kits (Millipore, Billerica, MA, USA and Diagnostic Systems Laboratories, Webster, TX, USA). Total cholesterol (TC), high-density lipoprotein cholesterol (HDL-C), low-density lipoprotein cholesterol (LDL-C), triglycerides (TRG), and blood glucose (GLU) were assessed using the Cholestech LDX blood analysis system (Cholestech, Hayward, CA, USA). Insulin sensitivity was estimated with homeostasis model assessment-estimated (HOMA-IR) as previously described [10].

### 2.9. Resting Energy Expenditure (REE), Heart Rate, and Blood Pressure

On laboratory testing days (week 0 and 17), resting energy expenditure (REE) was measured via indirect calorimetry using the ventilated hood technique (ParvoMedics; analyzed via True One software) as previously described [4]. Participants arrived at the Human Performance Laboratory immediately upon waking (between 0600 and 0730). Following 20 min of quiet lying, REE was measured for 30 min while subjects lay supine in a darkened, temperature controlled room. Only the last 25 min were used for calculation of the REE. Resting heart rate was recorded with telemetry (Polar Electro) and blood pressure was obtained with a standard mercury sphygmomanometer and stethoscope in the supine position following a minimum of 10 min of quiet resting; all measurements were obtained by the same investigator (PJA).

### 2.10. Internet-Based Healthy Lifestyle

Participants in both groups were provided a unique login and password to a website containing detailed content on relevant exercise and nutrition lifestyle strategies on a weekly basis. Specifically, each week emphasized a different component of the exercise routines and nutritional intervention they were to follow over the course of the 16 weeks study. This internet-based program served as a complement to the content delivery of the interventions by the investigators. However, all exercise routines were closely monitored and administered by at least two members of the research team. Weekly healthy lifestyle strategies provided through the web-based program included: stress reduction techniques; monitoring exercise intensity; protein-pacing; portion control; nutrient density and quality; meal/menu planning; breathing techniques; nutrient timing; non-exercise activity thermogenesis; healthy carbohydrates; phytonutrients; juicing; healthy fats, sleep quality; and supplementation.

### 2.11. Statistical Analysis

Statistical analyses were performed using SPSS software (version 21; IBM-SPSS, Armonk, NY, USA). A 2 by 2 factor repeated measures ANOVA (group; WP *vs.* FP X time; pre *vs.* post) was used to determine differences between groups and time points. Post-hoc comparisons were analyzed using Tukey’s test. Statistical significance was set at *p* < 0.05 for all analyses; and all values are reported as means ± SE.

## 3. Results

### 3.1. Participants and Compliance

A total of 30 individuals (*n* = 15 women; *n* = 15 men) were randomized into the interventions. A total of nine participants were removed from statistical analysis due to either non-compliance (<80% compliance) with whey protein supplementation (WP, *n* = 3), adverse cardiometabolic event unrelated to the study (FP, *n* = 3) and/or pre-existing injury (FP, *n* = 3). As such, twenty one participants (Table 1) completed the 16 week intervention (whey protein, WP, *n* = 9; food protein, FP, *n* = 12) and results are shown for all outcome measures.

### 3.2. Physical Performance Assessments

**Figure 2:**
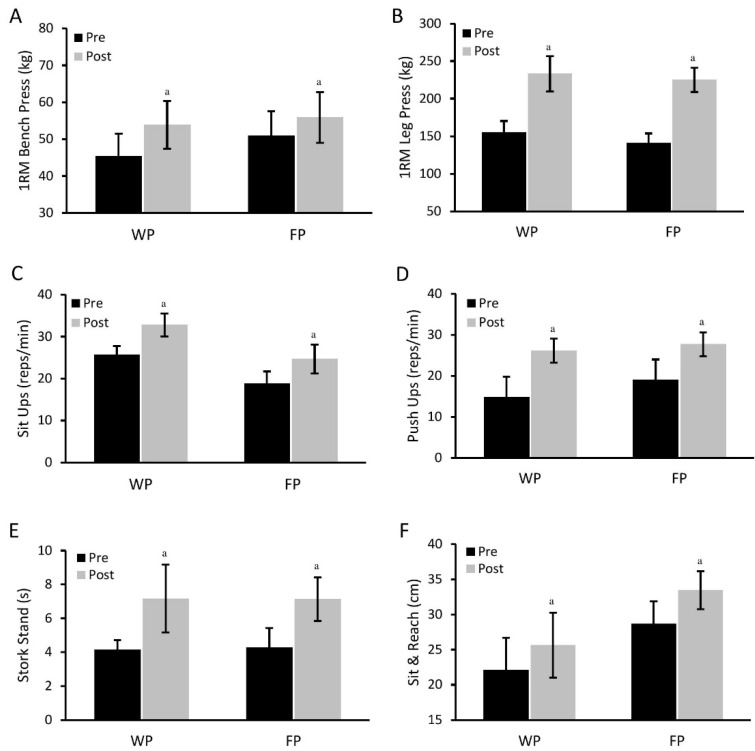

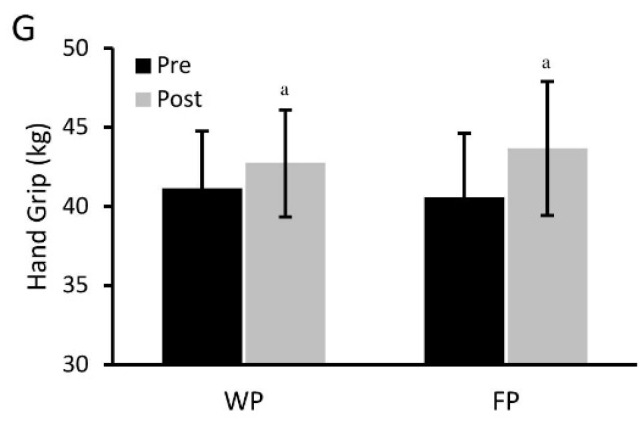
Comparisons of physical performance assessments between the food protein (FP) and the whey protein (WP) groups following the protein-pacing, resistance, interval, stretching, endurance training (PRISE): upper (**A**) and lower body maximal strength (**B**); core (**C**) and upper body muscular endurance (**D**); balance (**E**); flexibility (**F**) and grip strength (**G**). ^a^ Significantly different from baseline in each group (*p* < 0.01).

All physical performance measures improved significantly (*p* < 0.01) for each group following the RISE exercise protocol. In particular, upper and lower body maximal strength were significantly improved (*p* < 0.01, Figure 2A,B), with no group differences reported. Likewise, core and upper body muscular endurance were improved (*p* < 0.01, Figure 2C,D) following the RISE exercise protocol. Balance, flexibility and grip strength were also improved (*p* < 0.01, Figure 2E–G) with no differences between groups observed.

### 3.3. Body Weight and Composition and Resting Energy Expenditure

Significant improvements were observed across all measures of body composition after the 16 week intervention, though no differences were reported between groups (Table 2).

**Figure 3:**
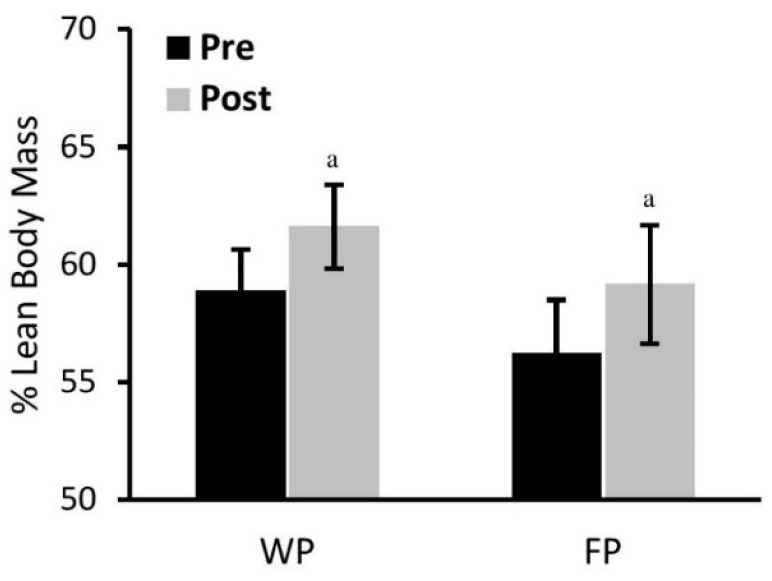
Comparison of percent lean body mass percent (%) lean body mass between the food protein (FP) and the whey protein (WP) groups following the PRISE training. ^a^ Significantly different from baseline in each group (*p* < 0.01).

Of particular interest was the significant improvement (*p* < 0.01) observed in percent lean body mass following the RISE exercise protocol in both groups (WP, 2.7%; FP, 2.9%; Figure 3). Resting energy expenditure (kcal/kg BW) showed a tendency (*p* = 0.10) to decline in both groups (WP; pre 18.9 ± 0.5 *vs.* post 18.1 ± 0.3: FP; pre 18.5 ± 0.7 *vs.* post 17.8 ± 1.0).

### 3.4. Cardiometabolic Markers

A significant improvement (*p* < 0.01) was seen in systolic blood pressure for each group, while resting heart rate and diastolic blood pressure did not change following the training intervention for either group (Table 3).

A significant improvement was observed in cholesterol, low-density lipoprotein, glucose, insulin, leptin, and adiponectin (*p* < 0.01, Table 3) following the RISE protocol, with no group effect reported. A significant improvement (*p* < 0.01, Table 3) was also reported for triglycerides, and to a significantly greater extent in the WP group (interaction, *p* < 0.05, Table 3). However, the group interaction observed was likely a result of the WP group having significantly higher triglycerides at baseline. The exercise training protocol did not significantly influence high-density lipoprotein or HOMA-IR in either group (Table 3).

### 3.5. Dietary Intake and Self-Reported Feelings of Hunger, Desire to Eat, and Satiety

All participants met recommended daily intakes at baseline, with no difference between groups (Table 4).

By design, each group significantly increased (*p* < 0.01) protein consumption in absolute (WP, 92 *vs.* 150; FP, 94 *vs.* 140 g/day) and relative amounts (WP, 17% *vs.* 33%; FP, 20% *vs.* 30%: WP, 1.0 *vs.* 1.7; FP, 0.9 *vs.* 1.6 g/kg BW/day). In contrast, carbohydrate intake significantly reduced (*p* < 0.05) in absolute (WP, 253 *vs.* 178; FP, 214 *vs.* 158 g/day) and relative (WP, 48% *vs.* 38%: FP, 43% *vs.* 34%) amounts in both groups. There was no significant change in the intake of fat, fiber, and omega 3 for either group (Table 4). Self-reported feelings of hunger decreased significantly in each group (*p* < 0.05), while feelings of satiety significantly increased (*p* < 0.05, Table 4). Subjective ratings for the desire to eat remained unchanged following the intervention.

## 4. Discussion

The primary purpose of the current study was to compare the effects of a protein-pacing diet consisting of either whey protein supplementation (WP; consumed as three of the six daily meals) or food protein from protein-rich food sources (FP; consumed for all six daily meals) combined with RISE training on fitness-related performance outcomes, as well as cardiometabolic and body composition measures. The main findings of this study were that increased dietary protein from either WP or FP combined with RISE exercise training for 16 weeks improved: (1) physical performance outcomes (upper and lower body maximal strength and endurance, flexibility, balance, and handgrip strength) and; (2) body composition (weight, waist circumference, body fat percentage, abdominal fat, visceral fat, and lean mass) and cardiometabolic markers (systolic blood pressure, blood glucose, LDL, total cholesterol, adiponectin) in both groups. Collectively, these results demonstrate, for the first time, increased dietary protein (>30% of total calories; >1.6 g/kg BW/day) from either WP or FP combined with the multimodal RISE protocol improves physical performance outcomes necessary for engaging in an active lifestyle, as well as enhanced cardiometabolic health in obese/overweight adults. Additionally, sources of dietary protein (whey *vs.* whole food) do not appear to be important factors determining such improvements.

### 4.1. Physical Performance

Diminished physical performance is a common consequence of aging, which may interfere with the ability to perform daily life activities [24]. Such diminution can, however, be reversed with exercise training in combination with increased protein consumption [1]. Despite such knowledge, little is known regarding the effects of lifestyle interventions combined with increased protein consumption on physical performance outcomes. Interestingly, we have recently demonstrated that the combination of the RISE training and whey protein supplementation is more effective in improving body composition and health outcomes than traditional resistance training, although how such improvement may influence physical performance still remains unclear [13].

In the present study, we extended our previous findings by demonstrating that the RISE protocol (16 weeks) combined with varying sources of protein consumption (>1.6 g/kg BW protein/day as five to six meals/day and >0.4 g/kg BW/meal) was effective in improving aspects of physical performance (upper and lower body maximal strength and endurance, flexibility, balance, and handgrip strength) in middle-aged overweight/obese adults (Figure 1). These findings are consistent with current recommendations [22] and previous studies investigating protein consumption and physical performance in healthy adults [20,23,25,26]. Interestingly, we have shown in the present study that whole food protein sources appear to be as effective in improving physical performance as whey protein supplementation, suggesting that increased protein consumption, irrespective of dietary sources, may improve physical performance when combined with exercise training. Our findings suggest that lean whole food protein sources (both animal and plant) are equally effective as whey protein to support body composition and physical performance outcomes in overweight/obese adults engaged with a multi-mode exercise program. Research has consistently shown whey protein’s ability to induce rapid absorption kinetics and the ability to stimulate muscle protein synthesis [27,28,29]. Thus, our finding of similar benefit from consuming lean, whole food protein sources is noteworthy, given the relatively high cost of protein supplementation.

The mechanisms underlying the improved physical performance are likely due to enhanced muscle protein synthesis and reduced protein breakdown [30]. A high plasma concentration of essential amino acids following protein consumption has been shown to work synergistically with the anabolic effect of exercise training (*i.e.*, resistance exercise) to enhance muscle protein synthesis and reduce protein breakdown [30]. In the present study, we utilized the RISE training protocol instead of traditional resistance exercise training based on our previous finding demonstrating its superiority in improving cardiometabolic health compared to traditional resistance training [13]. Collectively, our data suggest that a combination of a high protein diet consumed as five to six meals per day of >0.4 g/kg BW per meal (~32% of total kcals from protein), as either whey protein or lean whole food sources, and the RISE training protocol, are equally effective at improving physical performance outcomes in overweight/obese adults.

### 4.2. Body Composition

We found similar improvements in body composition, including reductions in weight, waist circumference, body fat percentage, abdominal fat, visceral fat, as well as an increase in percent lean mass (Table 2). It is noteworthy that such improvements occurred regardless of sources of dietary protein, suggesting similar effectiveness of increased protein consumption and exercise training in enhancing body composition. The mechanisms for the increased lean body mass are likely attributable to increased muscle protein synthesis and reduced protein breakdown [30]. In addition, the reduction in total and abdominal fat mass may be induced by enhanced subcutaneous and whole-body lipolysis, resulting in an increase in fat oxidation [31], as well as by enhanced energy expenditure [4].

Our findings are consistent with our previous study [13] that showed reductions in total and abdominal fat mass and an increased lean mass in overweight adults following the RISE training with whey protein supplementation (an addition of 60 g of protein to their daily diet). Most studies document a favorable increase in lean body mass following increased dietary protein (>1.5 g/kg BW/day) combined with exercise training [23], although this is not a universal finding [32]. Such discrepancies may be related to the amount and timing of protein intake. The strength of our study design was the matching of total protein intake in both groups (WP *vs.* FP) and an identical RISE exercise program performed by both groups throughout the 16 week intervention. This allowed us to systematically compare the source of increased dietary protein during exercise training on all outcome measures. Collectively, our findings support protein-pacing (five to six meals of 0.4 g/kg BW of protein) of both whey and food sources combined with multi-mode exercise training (RISE) to improve body composition in middle-aged overweight adults.

### 4.3. Cardiometabolic Biomarkers

The relationships between obesity, high blood pressure, and dyslipidemia have been well documented [33,34]. Increased adiposity is associated with the release of pro-inflammatory cytokines, leading to vascular wall injuries, arterial stiffening, blood pressure increases, atherosclerosis [35,36], and impaired glucose and fat metabolism [37,38]. Other important factors released from adipocytes are leptin and adiponectin, which play a role in the regulation of insulin sensitivity and body composition [39].

While these factors may be affected by increased levels of adiposity, previous studies have demonstrated that protein supplementation and exercise training may favorably combat obesity-related cardiovascular and metabolic risks [13,19,40]. In the current study, there were similar reductions in systolic blood pressure, total cholesterol, low-density lipoprotein cholesterol, leptin, insulin, and blood glucose concentrations, as well as an increase in adiponectin concentrations in both groups (Table 3). It is important to note that baseline leptin levels were different between groups due to two outliers (100 ng/dL; >±2 SD of the FP group mean, ±12.4 ng/dL) in the FP group. In fact, omitting these two outliers resulted in similar baseline leptin levels (FP, 23.1 ± 4.1 *vs.* WP, 26.0 ± 4.2, ng/dL). The reductions in systolic blood pressure have been attributed to consumption of whey protein and/or isoleucine-tryptophan [41]. The favorable lipid and adipokine changes are likely related to the increased lipolysis following protein supplementation and increased levels of physical activity [28,31].

Although the whey protein group also exhibited a reduction in triglyceride concentration, this was due to the high baseline value compared with the food protein group, whose concentration (mean of 94.2 mg/dL at baseline and 104.4 mg/dL at post-training) was in a healthy range throughout the intervention.

### 4.4. Satiation and Hunger Ratings, and Dietary Intake

In the present study, both groups exhibited similar improvements in satiation and hunger ratings (Table 4), which corroborates earlier work from our laboratory [42] and others [43] showing increased satiety and reduced hunger with increased protein consumption. It is important to highlight the current study was not caloric-restricted but instead was *ad libitum*. The fact that study participants reported a significant reduction in total calorie intake despite self-reported feelings of increased satiety and decreased hunger at the end of the 16 week intervention provides compelling support to encourage protein-pacing as a public health initiative to combat obesity-related diseases and enhance cardiometabolic and body composition health.

There are several limitations to the current study that are noteworthy and warrant further explanation. First, because previous work documented that the RISE training combined with whey protein supplementation was more effective in improving body composition and cardiometabolic health than whey protein alone or combined with traditional resistance exercise training [13], we did not include a non-protein supplemented RISE group. However, by design, this was intentionally omitted due to the well-known increased protein requirements during exercise training, particularly with obese, sedentary adults embarking on an exercise intervention. Thus, the current design afforded us the greatest degree of scientific rigor and rationale, as well as statistical control. Finally, our results should be interpreted and extrapolated to other ethnicities with caution as our cohort was predominantly white.

## 5. Conclusions

The current study demonstrates that a high protein diet from WP or from FP (>30% total calories from dietary protein; five to six meals per day (>1.6 g/kg BW/day or 0.4 g/kg BW protein/meal) combined with RISE training are equally effective in improving physical performance outcomes (upper and lower body maximal strength and endurance, flexibility, balance, and handgrip strength), body composition (total and abdominal fat), and cardiometabolic health (systolic blood pressure, blood glucose, LDL, total cholesterol, adiponectin) in middle-aged overweight adults.

## Figures and Tables

**Table 1:** Participant Characteristics.

Variable	WP	FP
Sex (M/F)	4/5	7/5
Age (year)	48 ± 4	52 ± 1
Height (cm)	173 ± 3	172 ± 3
Weight (kg)	96 ± 3	97 ± 5
Body mass index	32 ± 2	33 ± 1

WP: whey protein; FP: food protein. All values are means ± SE.

**Table 2:** Body composition measures at baseline and post-intervention.

Variable	WP			FP		
	Pre	Post	Δ %	Pre	Post	Δ %
Body Mass (kg)	95.8 ± 6.1	90.9 ± 5.5 ^a^	−4.8 ± 1.2	96.9 ± 4.8	92.2 ± 4.4 ^a^	−4.8 ± 0.9
Waist (cm)	105.2 ± 3.7	94.3 ± 3.0 ^a^	−10.1 ± 1.9	104.7 ± 3.1	96.3 ± 3.2 ^a^	−7.9 ± 1.3
Fat Mass (kg)	35.8 ± 2.9	31.7 ± 2.6 ^a^	−10.9 ± 2.3	38.4 ± 2.8	34.1 ± 2.8 ^a^	−11.9 ± 1.8
% Fat Mass	38.8 ± 1.9	36.2 ± 1.8 ^a^	−6.8 ± 1.6	41.4 ± 2.4	38.5 ± 2.6 ^a^	−7.5 ± 1.2
AbFat Mass (kg)	5.1 ± 0.4	4.2 ± 0.3 ^a^	−15.3 ± 4.7	5.5 ± 0.4	4.7 ± 0.4 ^a^	−15.9 ± 2.8
VAT (g)	1232.6 ± 219.9	884. 9 ± 173.3 ^a^	−29.9 ± 5.6	1816.8 ± 262.2	1498.2 ± 221.9 ^a^	−18.1 ± 4.8

Data presented as means ± SE. Pre, baseline; AbFat, abdominal fat; VAT, visceral adipose tissue. ^a^ Significantly different from baseline (*p* < 0.05).

**Table 3:** Cardiometabolic markers at baseline and post-intervention.

Variable	WP		FP	
	Pre	Post	Pre	Post
HR (beats/min)	63.3 ± 4.0	61.1 ± 2.1	64.8 ± 2.8	61.0 ± 2.3
SBP (mmHg)	130.4 ± 5.0	116.0 ± 4.1 ^a^	124.3 ± 3.3	119.2 ± 2.9 ^a^
DBP (mmHg)	83.6 ± 2.7	80.3 ± 2.3	84.8 ± 2.4	84.0 ± 2.1
TGL (mg/dL)	171.8 ± 29.8	123.2 ± 27.0 ^a^	94.2 ± 8.5 *	104.4 ± 12.8 ^#^
Cholesterol (mg/dL)	192.2 ± 20.0	151.3 ± 10.9 ^a^	197.8 ± 8.3	166.3 ± 5.0 ^a^
HDL (mg/dL)	42. 6 ± 4.0	46.1 ± 4.6	52.8 ± 4.6 *	50.0 ± 4.7 ^#^
LDL (mg/dL)	115. 2 ± 20.1	92.0 ± 9.6 ^a^	126.2 ±7.8	98.9 ± 5.1 ^a^
GLU (mg/dL)	97. 0 ± 4.6	93.6 ± 4.2 ^a^	104.0 ± 3.0	94.1 ± 2.0 ^a^
Insulin (μg/dL)	21.7 ± 10.5	7.5 ± 2.0 ^a^	9.6 ± 2.3 *	7.7 ± 2.0 ^a^
HOMA-IR (units)	1.0 ± 0.3	1.0 ± 0.3	0.9 ± 0.2	1.2 ± 0.3
LEP (ng/dL)	26.0 ± 4.2	18.7 ± 1.9 ^a^	42.4 ± 10.5 *	29.8 ± 9.3 ^a^
ADI (μg/dL)	18.5 ± 1.5	21.1 ± 1.5 ^a^	19.2 ± 1.6	23.5 ± 2.4 ^a^

Data presented as means ± SE. Pre, baseline; HR, heart rate; SBP, systolic blood pressure; DBP, diastolic blood pressure; TGL, plasma triglycerides; GLU, fasting plasma glucose; Insulin, fasting plasma insulin; HOMA-IR, homeostasis model assessment-estimated insulin resistance; LEP, leptin; ADI, adiponectin. ^a^ Significantly different from baseline (*p* < 0.05); * significantly different from WP at baseline (<0.05); ^#^ significantly different from WP post-intervention (<0.05).

**Table 4:** Dietary intake and hunger ratings at baseline and post-intervention.

Variable	WP		FP	
	Pre	Post	Pre	Post
Kcal	2146 ± 175	1889 ± 148 ^a^	2094 ± 194	1833 ± 158 ^a^
Protein (g)	92 ± 10	150 ± 14 ^a^	94 ± 7	140 ± 8 ^a^
Protein (%)	17 ± 2	33 ± 2 ^a^	20 ± 1	30 ± 2 ^a^
Protein (g/kg)	1.0 ± 0.12	1.7 ± 0.17 ^a^	0.9 ± 0.1	1.6 ± 0.2 ^a^
Carbohydrates (g)	253 ± 20	178 ± 19 ^a^	214 ± 17	158 ± 16 ^a^
Carbohydrates (%)	48 ± 2	38 ± 2 ^a^	43 ± 2	34 ± 2 ^a^
Fat (g)	77 ± 9	62 ± 4	75 ± 9	76 ± 12
Fat (%)	33 ± 2	30 ± 1	33 ± 2	36 ± 3
Fiber (g)	14 ± 1	26 ± 3	22 ± 3	23 ± 4
Omega 3	2 ± 0.5	2 ± 1	1 ± 0.1	2 ± 0.5
Hunger	36 ± 8	31 ± 7 ^a^	46 ± 6	30 ± 8 ^a^
Satiety	23 ± 7	44 ± 5 ^a^	37 ± 6	44 ± 11 ^a^
Desire to eat	39 ± 10	38 ± 7	48 ± 6	31 ± 6 ^a^

Data presented as means ± SE. Pre, baseline; Post, post-intervention. ^a^ Significantly different from baseline (*p* < 0.05).

## Supplementary Materials

The following are available online at http://www.mdpi.com/2072-6643/8/5/288/s1, Table S1: Sample Menus from the FP and WP nutritional intervention diet plans during the 16 week PRISE intervention. Menus were similar in macronutrient distribution, Table S2: RISE exercise training protocol.

## Abbreviations

PRISE	protein-pacing, resistance, interval, stretching, endurance training
WP	whey protein
FP	food protein
VAT	visceral adipose tissue
TC	total cholesterol
HDL-C	high density lipoprotein cholesterol
LDL-C	low density lipoprotein cholesterol
TRG	triglycerides
GLU	glucose
REE	resting energy expenditure
