# Supplementary material for: Protein-Pacing from Food or Supplementation Improves Physical Performance in Overweight Men and Women: The PRISE 2 Study
Source: Nutrients. 2016 May 11;8(5):288. doi: 10.3390/nu8050288 (PMC4882701; doi:10.3390/nu8050288)
Supplement: Supplementary file 1 [file nutrients-08-00288-s001.docx]

Supplementary Materials: Protein Intake from Food or Supplementation Improves Physical Performance in Overweight Men and Women: The PRISE 2 Study

Paul J. Arciero, Rohan C. Edmonds, Kanokwan Bunsawat, Christopher L. Gentile,
Caitlin Ketcham, Christopher Darin, Mariale Renna, Qian Zheng, Jun Zhu Zhang and
Michael J. Ormsbee

**Table S1.** Sample Menus from the FP and WP nutritional intervention diet plans during the 16 week PRISE intervention. Menus were similar in macronutrient distribution.

|  | **Food Protein (FP)** | **Whey Protein (WP)** |
| --- | --- | --- |
| ***Breakfast*** | Eggs/egg whites, steel cut oats, olive oil, honey, nut/seed butter, milk, fruit, tea/coffee  25 g protein; 30 g carbohydrate; 15 g fat | Eggs/egg whites, steel cut oats, olive oil, honey, nut/seed butter, milk, fruit, tea, coffee  25 g protein; 30 g carbohydrate; 15 g fat |
| ***Mid-morning snack*** | Food Protein Snack: Greek yogurt, fresh fruit, nuts  20–25 g protein; 20 g carbohydrate; 5 g fat | Whey Protein Shake: 1 scoop whey protein, fresh fruit, nuts  20–25 g protein; 20 g carbohydrate; 5 g fat |
| ***Lunch*** | Fish/poultry/beef, spinach, olive oil, honey mustard, baby carrots, bell peppers, dried cranberries, diced fruit, whole grain pita  25 g protein; 30 g carbohydrate; 15 g fat | Fish/poultry/beef, spinach, olive oil, honey mustard, baby carrots, bell peppers, dried cranberries, diced fruit, whole grain pita  25 g protein; 30 g carbohydrate; 15 g fat |
| ***Mid-Afternoon snack*** | Food Protein Snack: Cottage cheese, fresh vegetables, nuts  20–25 g protein; 20 g carbohydrate; 5 g fat | Whey Protein Shake: 1 scoop whey protein, fresh vegetables, nuts  20–25 g protein; 20 g carbohydrate; 5 g fat |
| ***Dinner*** | Fish/poultry/beef, whole grain rice/pasta or legumes, field greens, tomato, broccoli, chopped nuts, dried fruit, olive oil, milk  25 g protein; 30 g carbohydrate; 15 g fat | Fish/poultry/beef, whole grain rice/pasta or legumes, field greens, tomato, broccoli, chopped nuts, dried fruit, olive oil, milk  25 g protein; 30 g carbohydrate; 15 g fat |
| ***Evening snack*** | Food Protein Snack: Greek yogurt/cottage cheese, fruit, nuts  20–25 g protein; 20 g carbohydrate; 5 g fat | Whey Protein Shake: 1 scoop whey protein, fresh fruit, nuts  20–25 g protein; 20 g carbohydrate; 5 g fat |

**Table S2.** RISE exercise training protocol.

| **Exercise** | **Type** | **Work** | **RPE** | **Monday** | **Tuesday** | **Wednesday** | **Thursday** | **Friday** | **Saturday/Sunday** |
| --- | --- | --- | --- | --- | --- | --- | --- | --- | --- |
| Resistance (R) | WB | 2–3 sets/exercise  10–15 reps | 7–9 | WB | - | RECOVERY | - | - | RECOVERY |
| Intervals (I) | C | 7–10 sets  30–60 s work  2–4 min rest | 10/3 | - | X |  | - | - |  |
| Stretching (S) | S | ≤60 min | 7–8 | - | - |  | WB | - |  |
| Endurance (E) | C | ≥60 min | 6 | - | - |  | - | X |  |

RPE, rating of perceived effort; C, choice of exercise modality; WB, whole body exercise; S, stretching exercise; X, exercise day. Exercise modalities available for C include: running, cycling, swimming, elliptical, rowing, cross-country skiing, *etc*.
